# Demographic Consequences of Poison-Related Mortality in a Threatened Bird of Prey

**DOI:** 10.1371/journal.pone.0049187

**Published:** 2012-11-14

**Authors:** Simone Tenan, Jaume Adrover, Antoni Muñoz Navarro, Fabrizio Sergio, Giacomo Tavecchia

**Affiliations:** 1 Population Ecology Group, Mediterranean Institute for Advanced Studies (Consejo Superior de Investigaciones Científicas–University of the Balearic Islands), Esporles, Mallorca, Spain; 2 Sezione Zoologia dei Vertebrati, Museo delle Scienze, Trento, Italy; 3 Dipartimento di Scienze della Terra e dell'Ambiente, Università di Pavia, Pavia, Italy; 4 Grup d'Ornitologia Balear i defensa de la naturalesa, Palma, Mallora, Spain; 5 Department of Conservation Biology, Estacion Biologica de Doñana - CSIC, Seville, Spain; University of Bern, Switzerland

## Abstract

Evidence for decline or threat of wild populations typically comes from multiple sources and methods that allow optimal integration of the available information, representing a major advance in planning management actions. We used integrated population modelling and perturbation analyses to assess the demographic consequences of the illegal use of poison for an insular population of Red Kites, *Milvus milvus*. We first pooled into a single statistical framework the annual census of breeding pairs, the available individual-based data, the average productivity and the number of birds admitted annually to the local rehabilitation centre. By combining these four types of information we were able to increase estimate precision and to obtain an estimate of the proportion of breeding adults, an important parameter that was not directly measured in the field and that is often difficult to assess. Subsequently, we used perturbation analyses to measure the expected change in the population growth rate due to a change in poison-related mortality. We found that poison accounted for 0.43 to 0.76 of the total mortality, for yearlings and older birds, respectively. Results from the deterministic population model indicated that this mortality suppressed the population growth rate by 20%. Despite this, the population was estimated to increase, albeit slowly. This positive trend was likely maintained by a very high productivity (1.83 fledglings per breeding pair) possibly promoted by supplementary feeding, a situation which is likely to be common to many large obligate or facultative European scavengers. Under this hypothetical scenario of double societal costs (poisoning of a threatened species and feeding programs), increasing poison control would help to lower the public cost of maintaining supplementary feeding stations.

## Introduction

The current rate of biodiversity loss has generated concerns on the future of many wild populations and increased the need for population monitoring and risk assessment. The expected long-term trend of a population and its probability of extinction are typically obtained through population viability analyses [1]–[3]. The core of this analysis is a mathematical model that projects the current state of the population into the future and estimates population extinction, or quasi-extinction, probability. The population model, which includes survival and fertility parameters and projects the population state, is usually parameterised using estimates of demographic parameters derived from individual-based information, i.e. capture-recapture data [4]. Despite much effort to increase the precision and realism of capture-recapture analyses (e.g. [5], [6]), the use of a single dataset to parameterise the population model poses the problem of model validation [7]. The latter can be implemented by retrospective analyses [4], for example by comparing model predictions with population surveys [8], but this ignores sampling errors associated with counts [9], [10]. Also, the many sources of variation in large capture-recapture datasets generally violate the assumptions of population dynamics models (see [11], [12]), propagating errors into the estimate of extinction risk [13]. Recently various computational approaches have been proposed to integrate data from different sources of information, such as the P-system based models (e.g. [14], [15]) and integrated population models [10], [13], [16], [17]. In particular, the latter allows the incorporation of counts and individual-based data into a single analysis through a joint likelihood. In this integrated analysis population counts are linked to population state by an observation equation, while a state equation describes the link between population state and demographic processes through a population model (hereafter ‘transition model’) based on per-capita survival and fecundity taken from individual-based data [10]. The transition model is structurally similar to the one used in population projections but is constructed through parameters that integrate information simultaneously acquired from different sources. Such integrated models yield multiple advantages: (1) their integrated structure reduces parameter uncertainty [16]; (2) their consensual estimates increase the realism of population state forecasting and incorporate into model predictions the variance and covariance between different demographic parameters; and (3) they allow estimation of latent parameters, i.e. parameters that appear in the biological process, i.e. the population model, but not measured empirically [10]. At present, integrated modelling represents a useful extension of the classical analyses based on a single type of data. This is particularly evident in those cases where information on population threats is available for different data sources or at different spatial scales. For example, individual life-history data of long-lived seabirds come mainly from observations at the breeding colony, whereas the main threat to population persistence is the mortality at sea due to fishery by-catch [18]. Integrated modelling allows the integration of these two types of data that are typically analysed separately (see for example [18]–[20]). Similarly, Schaub et al. [17] integrate capture-recapture data of eagle owls *Bubo bubo* with the number of owls found dead on the roads and reported by the public.

This sort of analysis is ideally suited to the assessment of conservation threats to endangered organisms, such as many top predatory taxa. The charismatic nature of these species makes them the focus of attention and monitoring by multiple figures, including professional researchers, public administrations and amateurs, leading to simultaneous but heterogeneous sources of information. A good example in this context is offered by birds of prey, a group of species typically monitored by different entities and frequently subject to direct or indirect human related mortality such as illegal hunting [21], primary and secondary poisoning [22], habitat destruction [23], prey depletion, collision with windmills and electrocution on power lines [17], [24], [25]. Despite their legal protection in several countries, many raptor populations continue to be at risk, as showed by a worldwide deterioration of their conservation IUCN index [26]. Therefore, methods allowing optimal integration of available information to estimate the relative impact of human-related mortality on population growth could represent major advances in our capability to plan management and halt declines. To this aim, we offer an example of implementation of integrated modelling to estimate the impact of illegal poisoning on a threatened raptor. Illegal poisoning is a form of persecution usually generated by conflicts with human interests associated with livestock rearing or hunting, and indiscriminately affects birds or mammals that occasionally or regularly feed on carcasses, or other poison-soaked baits (e.g. pesticides as carbofuran or alpha-chlorolose; [22], [27]). Toxicoses related to this illegal activity have been identified as the main threat for the conservation of different species of raptors in Europe and Asia (e.g. [28]). Poison-related mortality often affects breeding adults, and several studies have documented the detrimental effects of this factor on population dynamics, especially for long-lived species with low reproductive rates and delayed maturity [29]. Our model species, the Red Kite *Milvus milvus* is a medium-sized raptor distributed exclusively in the western Palearctic [30]. Since the 19th century, the species has declined throughout Europe, and many of its populations are nowadays considered endangered due to the illegal use of poisoning baits to control predators of game species [21], [22], [31]. In Spain, which holds one of the largest breeding and wintering populations of Europe, Red Kites have been added to the red list of species at risk of extinction in 2011. On the 3,640 km^2^ island of Mallorca of the archipelago of Balearics (Spain), the population was reduced to only 7–8 pairs in the year 2000 [32]. The population has recently increased to 19 breeding pairs, but its small size makes it still vulnerable to stochastic peaks of adult mortality, such as those caused by poisoning. A previous analysis of intensive radio-tracking data from this population showed that illegal poisoning accounted on average for 53% of the mortality [6]. However, this estimate was based on marked birds only and its effect on the population growth rate is unknown. Our first aim was to obtain a more precise and ‘consensual’ estimate of mortality due to illegal poisoning. We did so by combining four different types of information: detailed monitoring on radio-marked birds, the number of breeding pairs from annual surveys, the number of fledglings, and the number of birds found poisoned and brought to the local rehabilitation centre by the public. These four data sources were mathematically combined into a population model incorporating the age-dependent demographic parameters. By combining separate datasets we were able to increase estimate precision and estimate the proportion of breeding birds, a parameter that was not directly measured in the field. We then used the consensual estimates derived from the integrated model to parameterised the age-structured population model and assess the demographic consequences of poison related mortality using perturbation analyses.

## Results

To verify the precision enhancement yielded by the integration of multiple datasets, we estimated three sets of parameters by integrating the information sequentially. The first set incorporates the individual data only (MS). The second integrates them with the survey of breeding pairs and the number of fledglings (IPM1), while the third adds to the previous sets the information on birds found dead and reported to the local rehabilitation centre by the general public (IPM2). As expected, the additional information resulted in increased parameter precision (Table 1). More specifically, precision was most improved for the estimate of the proportion of adult birds dying because of poison (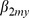, 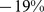 in the 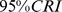) and for the reporting rates of birds found dead by poisoning without a functioning radio-tag (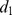, 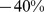). The difference between the mean estimates obtained from the full integrated model (IPM2) and those from IPM1 and MS was larger for the probability of encounter of dead animal without a functioning radio (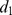, 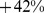; 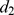, 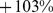).

**Table 1:** Estimated demographic parameters of the Red kite population of the island of Mallorca (Spain).

	IPM2			IPM1			MS		
Parameter	Mean	Lower	Upper	Mean	Lower	Upper	Mean	Lower	Upper
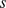	0.808	0.772	0.841	0.814	0.776	0.849	0.821	0.782	0.857
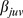	0.428	0.274	0.590	0.429	0.273	0.593	0.428	0.270	0.593
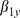	0.764	0.508	0.942	0.755	0.491	0.942	0.758	0.493	0.942
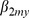	0.764	0.464	0.943	0.688	0.356	0.923	0.690	0.365	0.924
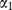	0.862	0.820	0.900	0.862	0.820	0.900	0.862	0.820	0.900
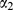	0.333	0.191	0.493	0.333	0.191	0.493	0.334	0.191	0.494
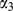	0.045	0.002	0.097	0.045	0.002	0.097	0.045	0.002	0.097
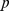	0.990	0.972	0.999	0.990	0.972	0.999	0.990	0.973	0.999
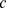	0.328	0.255	0.405	0.331	0.253	0.413	0.315	0.245	0.391
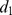	0.075	0.036	0.099	0.053	0.009	0.097	0.054	0.009	0.097
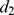	0.461	0.168	0.899	0.227	0.022	0.745	0.250	0.025	0.793
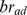	0.631	0.504	0.898	0.676	0.507	0.953	–	–	–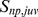	0.890	0.852	0.925	0.894	0.856	0.928	–	–	–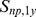	0.955	0.904	0.989	0.954	0.904	0.989	–	–	–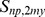	0.955	0.895	0.989	0.942	0.877	0.986	–	–	–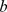	1.825	1.529	2.153	1.778	1.484	2.103	–	–	–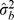	0.110	0.004	0.328	0.111	0.004	0.330	–	–	–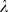	1.136	1.063	1.222	1.142	1.067	1.230	–	–	–

We show the posterior mean and 95% credible interval (95%CRI, lower and upper limit) of the estimates, obtained by a full integrated model (IPM2), an integrated model without considering data of unmarked birds found dead (IPM1), and a multi-state model with only radio-tracking data. For parameter notation see Methods.

The temporal variability of fecundity was slightly different from zero (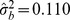, 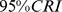: 0.004, 0.328), but the pattern of average fecundity, 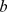, showed no obvious temporal trend.

In the transition matrix, we specified the proportion of breeding females older than 2 years, 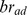, as a latent parameter, i.e. a parameter for which information was not available. The adult breeding proportion was difficult to estimate because birds loose the radio-transmitters when about four years old. This hidden, or latent, parameter was estimated on the basis of the remaining integrated information to be 0.63 and, despite a relatively large level of uncertainty, sensitivity analyses indicated that it was estimable (see Parameter estimation and model implementation subsection in Methods). The age-independent survival rate (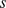) was 0.808 (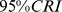: 0.772, 0.841). The probability of dying because of poisoning was age-dependent, being higher for birds older than one year (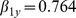, 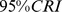: 0.508, 0.942; 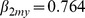, 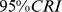: 0.464, 0.943) than for juveniles (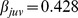, 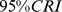: 0.274, 0.590). Assuming that human-related mortality was additive, survival probabilities in the absence of illegal poisoning can be derived from 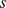 and each age-specific 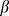 (eq. 13). Simulated, poison-free survival was higher in adult birds (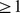 year old; 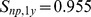, 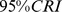: 0.904, 0.989; 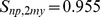, 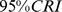: 0.895, 0.989) than in juveniles (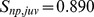, 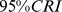: 0.852, 0.925). From these estimates we calculated that illegal poisoning reduced survival probability by 15% in adults (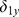 and 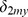) and 9% in juveniles (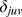).

**Figure 1:**
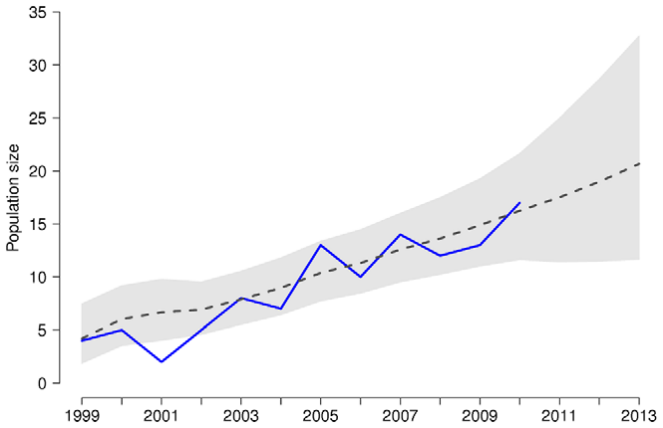
Observed and estimated sizes of the Red kite population of Mallorca (Spain), with a future projection of the number of breeding pairs. The blue solid line represents the surveyed population size, the black dashed line the predicted spring population sizes along with their 95%CRI (grey shading).

The smoothed estimates of the total annual number of breeding pairs, as well as the observed population sizes, showed a positive trend with a population growth higher than one (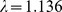, 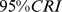: 1.063, 1.222) and roughly constant throughout the study (Fig. 1). Predictions of population size over the next three years (2011–2013) suggest a slow increase in the number of breeding pairs, although the 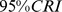 expands over time reflecting increasing uncertainty (Fig. 1).

**Figure 2:**
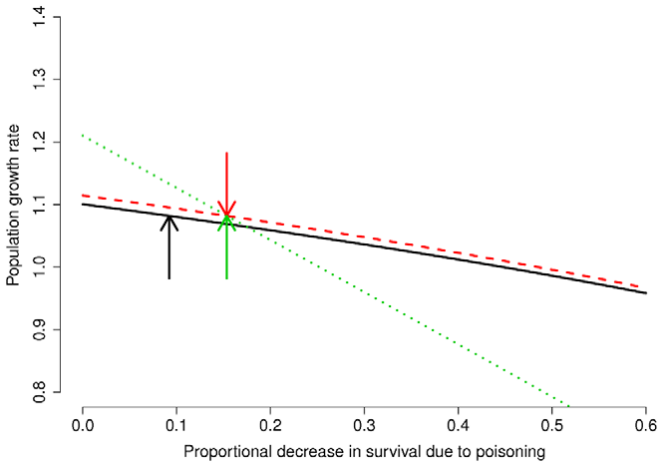
Changes of population growth rate in relation to changes in the proportional decrease of age-specific survival probability. The black solid line represents the relationship with proportional reduction in juvenile survival (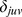), the red dashed line refers to 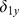, and the green dotted line refers to 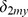. Current age-specific values of 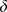 are indicated by the arrows with the same colour of the curve to which they refer.

**Figure 3:**
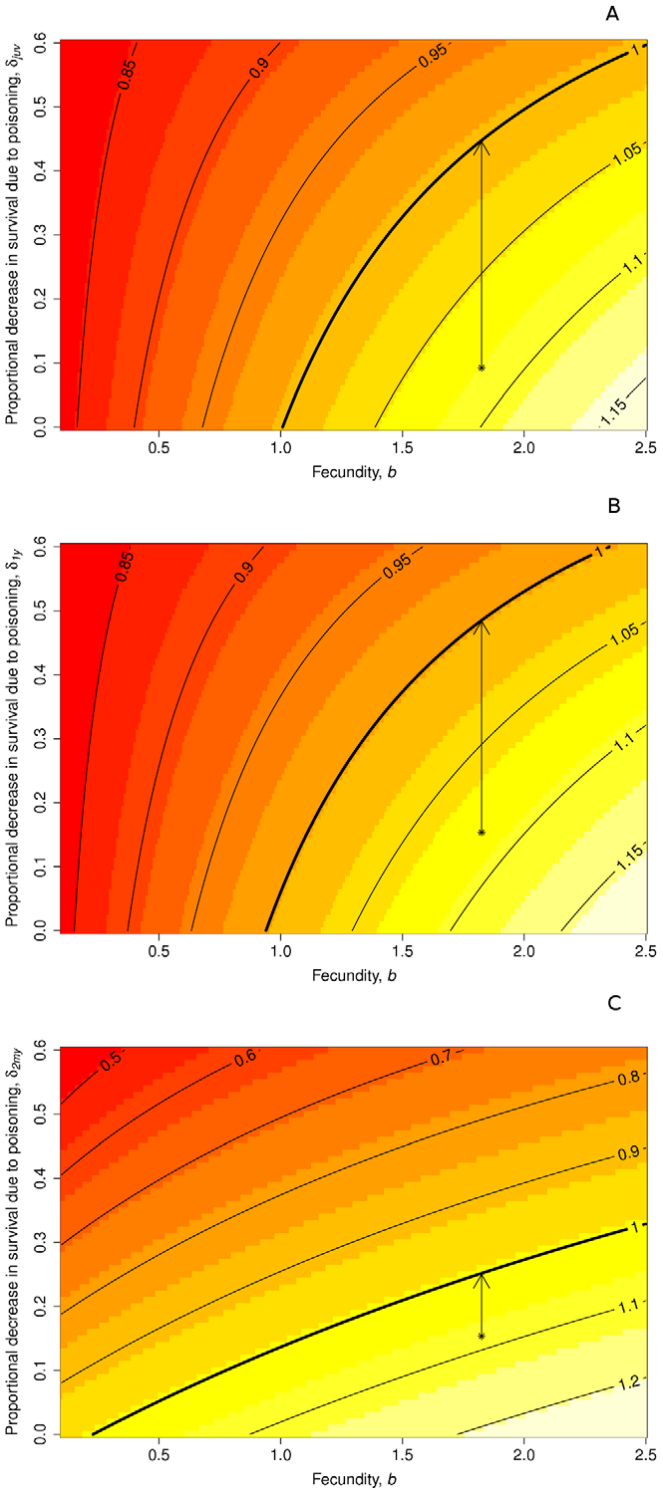
Difference between the proportional changes in age-specific survival probability due to illegal poisoning, fecundity, and population growth rate. The bold line represents population stability. The asterisks refer to the current parameter estimates, while arrows represent a theoretical increase in 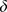 up to the level of population stability. a) juveniles. b) 1 year old. c) 2 or more year old red kites.

The combination of mean estimates of demographic parameters (IPM2, Table 1) in the deterministic model suggested a slightly positive population growth (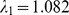) which matches well the slow increase in counts of breeding pairs. The population is thus projected to increase by 8.2% per year. When we explored how this rate was affected by an increase of survival probability due to lowered poisoning (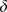), assuming that such mortality was additive, we found that the population would decline (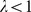) if survival will be reduced by 45%, 49%, and 25% for juveniles, 1 year old, and 2 or more years old individuals, respectively (that would lead to survival probabilities of 0.49, 0.49, 0.72; Fig. 2). Sensitivity of population growth to the different demographic rates was higher for adult survival in the absence of illegal poisoning (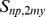) and the related proportional decrease due to this mortality cause (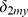; Table 2). The deterministic model indicated that a further reduction in survival probability, with 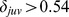, 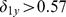, 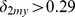 would not be compensated even by the maximum fecundity recorded for the species (2.2 young fledged per breeding pair [33]; Fig. 3).

**Table 2:** Sensitivity and elasticity of population growth rate of the Red kite population of the island of Mallorca (Spain).

Parameter	Sensitivity	Elasticity
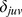	0.207	0.018
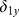	0.222	0.036
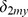	0.834	0.118
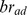	0.283	0.165
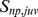	0.211	0.174
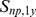	0.197	0.174
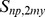	0.739	0.652
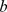	0.103	0.174

For parameter notation see Methods.

**Figure 4:**
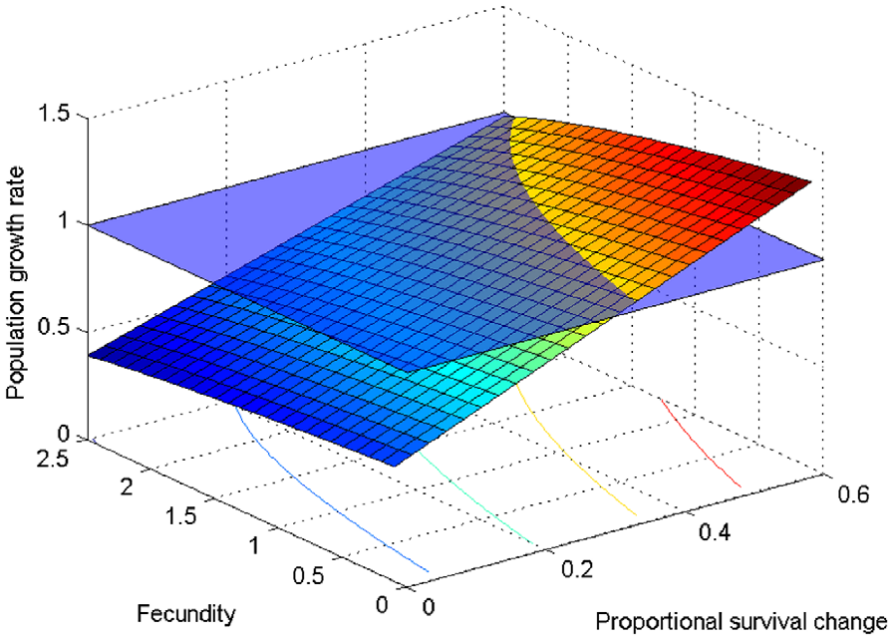
Age-independent relationship between population growth rate, fecundity, and proportional change in survival probability due to illegal poisoning. The blue horizontal plane represents population stability.

Finally, we explored an average age- and time-independent relationship between population growth rate, fecundity, and the proportional reduction in survival probability due to illegal poisoning. Results indicated that a decline in survival is proportionally more difficult to be compensated by an increase in per capita fecundity (Fig. 4).

## Discussion

### An analytical framework to help a ‘crisis discipline’

Conservation biology is often referred to as a crisis discipline because anthropogenic alterations and the rate of population extinctions ‘do not give the luxury of time’ [34], [35]. For this reason, diagnosis of population threats is often based on small sample size and evidence may come from scattered sources. As a consequence, marked uncertainty accompanies inferences on population trajectories or on the relative importance of different mortality causes. Here, we showed a statistical framework that increases estimate precision by analysing multiple data simultaneously. In particular, we integrated radio-tracking data, nest and fledglings counts and time series of birds found dead by the general public into a single analysis to obtain consensus estimates and explore the demographic impact of poisoning on an endangered raptor. The joint analysis delivered more precise estimates, which incorporated all available information and led to a more accurate assessment of the importance of specific causes of mortality and short-term population forecasting. Moreover, the integrated analysis allowed the estimation of the breeding and non-breeding sector of the population, an elusive parameter that can be measured only under special circumstances and usually through a pronounced survey (e.g. [36]). Finally, it is interesting to note that the raw frequency of animals reported to have died from a specific cause is an information frequently available from local authorities but generally considered as too coarse to yield any meaningful estimate of mortality impact. In our case, even if this information was scarce, it contributed to improve parameter estimates.

Information from multiples sources can be joined into a single analysis as long as data are independent, at least partially [16], [37]. The assumption of independence causes a trade-off between statistical needs and ecological realism. Indeed, joining independent data may help to meet model assumptions, but it increases the variance components, for example because separate information collected at different spatial scales may not be fully comparable. In our case, data came from an insular population, which minimises the noise related to immigration, emigration, or other sources of variance potentially included in the analyses when data are drawn from different populations. On the other hand, radio-tracked birds were only a portion of the total population and we considered only unmarked birds in the time series of kites reported dead by the public, in order to employ as independent datasets as possible.

### Illegal poison and population trajectories

The aim of this work was to measure the demographic consequences of illegal poisoning for a threatened raptor. The proportion of dead kites which were killed by poisoning in our study was considerable as it varied from 0.43 to 0.76 for yearlings and older birds, respectively. Smart et al. [21] reported a similar figure for yearlings in the UK, but a smaller value for older birds. Despite an increase of 15% in mortality probability due to poisoning, our population seems to be slowly growing and is expected to continue to increase in the immediate future. We assumed a constant sampling variance during the study period and, although unlikely, we think it is a reasonable assumption given the small number of breeding pairs each year. The positive trend is likely to be maintained by a very high productivity (one of the highest registered for the species, [21], [33], [38]), which seemed to compensate for small declines in adult survival (Fig. 3). The high productivity was probably promoted by the local presence of a rubbish dump (closed in 2009) and by supplementary feeding stations established by local authorities in recent years. Unfortunately, no specific data are currently available to measure the exact role of supplementary feeding in our population. The effectiveness of artificial feeding sites in improving productivity and sustaining population viability is still debated [39]–[42], for example Oro *et al.* [42] reported that feeding stations suppressed the dispersal of immature birds, which increased the competition among individuals and resulted in a negative impact on population growth. In our case, this mechanism seems unlikely because the population is not habitat-limited and we only know few cases of pre-breeding dispersal outside the study area. If supplementary food promoted population growth this could generate a paradoxical scenario where anthropogenic mortality is compensated by food supplemented by humans. Under this scenario of double societal costs, increasing poison control might help to lower the public cost of maintaining supplementary feeding stations. Finally, even though the population is slowly increasing, the number of breeding pairs is still small and we estimated that the population growth would be 20% higher without the additional mortality due to poisoning. Clearly, our analysis confirmed the extreme sensitivity of this extinction-threatened species to poisoning, as suggested by previous authors (e.g. [21], [22], [38]).

### Facultative, occasional and obligate scavengers and the illegal use of poison

As a facultative scavenger, the Red Kite is frequently attracted to small poisoned baits that are illegally used to control mammalian predators of game species. The demographic impacts outlined for our population are likely to apply to other occasionally scavenging raptors, such as Spanish imperial eagles *Aquila adalberti*, or to obligate scavengers such as bearded vultures *Gypaetus barbatus* or Egyptian vultures *Neophron percnopterus* (e.g. [27], [29], [42], [43]). In our case, perturbation analyses indicated that population growth was more sensitive to adult survival in the absence of poisoning (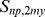) and to the related proportional decline due to this mortality cause (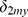). A similar outcome is expected for obligate scavengers, such as vultures, whose survival in the absence of poisoning is typically high [42], [44], [45]. In these species, characterized by extended longevity and low natality, the proportional decline in survival caused by poisoning is likely to have a stronger impact on population growth because their typically low fecundity cannot function as a buffering trait. Therefore, the scenario outlined for our population could be considered as a simulation of minimum impact, when compared to many larger, longer-lived and more obligate scavengers.

### Conservation measures

Our results suggest that illegal poisoning was the most important cause of mortality of Red Kites in the Island of Mallorca and was able to suppress population growth by 20%. Nevertheless, a high fecundity rate seemed helpful to counter-sustain population viability. Unfortunately, it is not possible to evaluate to what degree this could be ascribed to the beneficial effect of artificial feeding sites, and further research is needed to understand their role as a population management tool. As already stressed by other authors, future management activities should concentrate on the eradication of illegal poisoning [29], and on devising techniques of predator control compatible with the conservation of the Red Kite and other vulnerable species [27]. Improving the monitoring, surveillance and the post-mortem lab-analyses of illegal poisoning episodes could further help to build the necessary pressure to reduce the cases of malicious or negligent actions [46]. In this way, an accurate forensic diagnosis has a key role in the investigation of poisoning [47] and could be supported by techniques that facilitate rapid mortality detection, such as radio tagging and other remote sensing devices.

## Materials and Methods

### Data collection

The field data were collected from the Red Kite population of the island of Mallorca, in the archipelago of Balearic Islands (Spain). From 1999 to 2010, the whole island was intensively surveyed annually to count the number of breeding pairs. Territorial pairs were censused throughout the whole island and by visiting formerly occupied breeding sites during the spring courtship period. For our analysis we retained only the number of active nests where at least one egg was laid (hereafter breeding pairs). All broods were intensively monitored and each year the number of fledglings was recorded. Moreover, 142 red kites were equipped with VHF radio-tags (TW-3 model by Biotrack; lifespan: c. 3–4 years) just before fledgling during the period 2000–2010. Individuals were handled following rules and permissions by Conselleria d'Agricultura Medi Ambient i Territori of the Government of the Balearic Islands. In addition to radio-tags, all chicks were marked using PVC wing-tags with a unique alpha-numeric code, one on each wing. The wing-tags were used to assess the loss of radio signal ceased by mechanical or electrical failures. Simultaneous loss of both types of tags was never observed, and all dead birds, found with or without transmitters, had retained at least one wing-tag [6]. All tagged birds were searched monthly throughout the whole island by car or, occasionally, helicopter. Here, we retained the information on live resightings made from April to June only, whilst we gathered information on birds recovered dead throughout the year. All recovered carcasses were examined post-mortem to establish the cause of death. Exposure to a toxic substance was confirmed by toxicology analyses. Additional observations were obtained at feeding stations, territories and roost sites to record the presence of birds whose radio signal had been lost. Finally, we compiled the number of unmarked kites brought to the local wildlife rehabilitation centre between 1999–2010 and killed by poisoning or other causes. These two time-series were formed by a total of four poisoned birds and eleven individuals killed by causes other than poisoning (electrocution, aircraft collision, drowning in artificial water reservoir and other unknown causes). Recovered birds whose radio failed before death were discarded to avoid dependence with radio-tracking data.

### Integrated population model

The different sources of demographic information (population surveys, number of fledglings, radio-tracked birds and recoveries of dead individuals) were combined into a single model. The major advantage of analysing all data sets within a single model simultaneously is that the precision of parameter estimates is increased and parameters for which no explicit data are sampled can be estimated [16], [17], [48]–[54]. The integrated model was fitted in the Bayesian framework because this provides more flexibility than the frequentist framework and exact measures of parameter uncertainty [16], [51], [52].

### Likelihood for the population count data

To describe the model, we began by describing the likelihoods components for the different demographic parameters and subsequently defined how they would be linked and estimated in a single overall model. Survey data were modelled by a state-space model [48], which consisted of a set of states that described the true but unknown development of the population and an observation process linking the observed population counts to the true population size assuming an observation error [51], [52]. The state process was described deterministically by a female-based, pre-breeding matrix projection model [4] with three age classes (1, 2 and 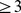 years old respectively) as
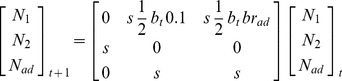
(1)where 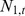 is the number of 1 year old females at time 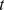, 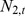 is the number of females of 2 years old at time 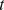, and 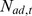 is relative to females older than 2 years at time 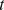. Survival probabilities of a female between time 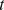 and 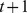 is denoted 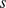, and 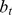 is the fecundity at time 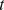. Although the model was female-based, fecundity refers to the complete reproductive output and it was halved to account for the number of females raised per breeding female. This was justified by the even sex ratio observed for a sub-sample of genetically sexed fledglings (J. Adrover *unpublished data*).

Based on intensive monitoring of radio-tagged birds, Red Kites usually begin breeding at 3 years old or later, although in Mallorca c. 10% of females bred in their second year of life [6]. However, the proportion of breeding females older than 2 years, 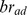, was estimated as a latent parameter, because it was impossible to obtain a figure from our own data due to the limited lifespan of the radio-tags (see below).

Since 1999 we are aware of only one case of emigration from Mallorca (from a sample of 230 marked birds). As a consequence we assumed that no immigration or permanent emigration from the island were present (J. Adrover *unpublished data*).

To account for demographic stochasticity, we used Poisson and binomial distributions to describe the dynamics of the true population size over time, already described by the population model in eq. 1. Specifically, the age-specific numbers of females in year 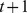 were modelled as
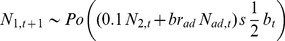
(2)

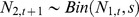
(3)

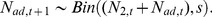
(4)The observation process is conditional on the state process. We assumed the counts of breeding females in year 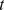 (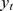) to follow a Poisson distribution [51], [52], [55] as
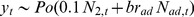
(5)The likelihood of the population count data is 0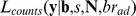.

### Likelihood for radio-tracking data

We implemented the model outlined in [6] as a multi-state capture-recapture model in a Bayesian framework [51], to estimate an age-independent survival probability, the age-dependent mortality of marked birds, the incidence of tag loss and the relative magnitude of different sources of mortality.

The observation of live and dead birds, together with the information on tag loss, formed the set of observable events from which we estimated the proportion of birds that died by poisoning or by other (natural) causes. We considered that individuals can move across three main states: alive, dead by poison, and dead because of other causes. Given that individuals can lose their radio transmitter, we considered the above states for birds with and without a functioning radio. Moreover, we included an additional dead state that corresponds to an unobserved dead state [51], [56]. Therefore, observable “recently dead” individuals move to state “unobserved dead” at the next occasion. This latter state is absorbing, meaning that once individuals are in this state they will remain there [51]. This differentiation assumes that corpses are found soon after death and allows us to estimate the reporting rate associated with the observable dead states and the probability of dying from different causes [57]. For alive birds we distinguished six age classes: juveniles (noted ‘juv’) spanning the time from tagging as nestlings up to the end of the first year of life, one-year olds (‘1y’) to the time between 1 and 2 yr old, two-year olds (‘2y’) between 2 and 3 years old, three-year olds (‘3y’) between 3 and 4 years old, four-year olds (‘4y’) between 4 and 5 years old, and five or more year olds (‘5my’) from 5 yr old to all following years. We considered six age-specific states in relation to lifespan of tag batteries, that did not exceed 3–4 years. A marked bird may survive from year 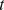 to year 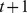 with probability 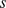, or it may die with probability 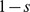 some time during the year. If it dies, this is either because of poisoning with probability 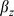 (subscript 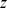 refers to the following age-classes, 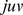, juvenile; 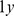, one year old; 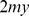, 2 years or older) or because of any other cause with probability 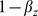. The fate of a marked individual also accounts for the radio signal retention 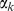. The subscript 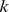 define three age-classes on the basis of radio signal decay probability, as estimated in [6] (1 to the time between tagging as nestlings up to the end of the third year of life, 2 between 3 and 4 years old, 3 for all the following years).

**Figure 5:**
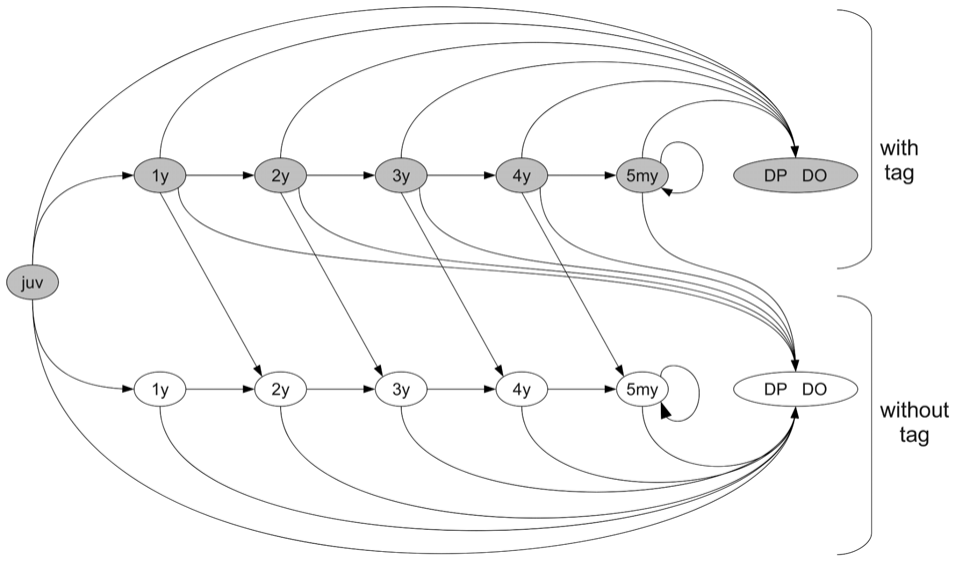
Diagram of possible states of a marked red kite. Transitions between two subsequent states, from time 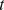 to 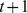, are denoted with arrows and correspond to parameters in the transition matrix of Fig. S1. For the sake of clarity, the parameters and the “unobserved dead” state are not reported. Notation: DP: dead by poison; DO: dead by other causes.

The above states, in relation with tag retention and different age classes lead to a 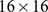 transition matrix available as supporting information (Fig. S1). Between any interval, individuals might change state according to the transitions in Fig. 5.

At any given time, we could observe 16 types of mutually exclusive events, arbitrary coded with numbers from 1 to 16 (Fig. S2). The events coded from ‘1’ to ‘6’ refer to encounters of individuals alive with a functioning radio and belonging to one of the six age-classes mentioned above. Similarly, codes from ‘9’ to ‘13’ refer to birds alive and without a functioning radio. Note that the latter codes are referred only to the five age-classes of non-juveniles birds. Codes ‘7’ and ‘14’ refer to individuals found poisoned with and without a functioning radio respectively. Similarly, ‘8’ and ‘15’ code for birds found dead for causes other than poisoning, with and without a functioning radio respectively. These codes do not distinguish whether the radio was physically lost or not functioning. The last possible event (coded ‘16’) refers to cases when the radio signal cannot be heard and the animal cannot be seen. This may correspond to any underlying state: for example, the animal may have lost the radio or be carrying one that ceased to function, or it may be dead having lost the radio and remaining undetected. Each of the other events can happen only with one state.

Conditional on the different fates, an animal with a functioning radio may be encountered with probability 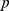, while an animal without radio signal may be encountered with probability 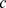 if alive, 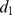 if dead by poisoning, and 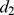 if dead by other causes.

The likelihood of this sub-model is categorical and we used a state-space parameterization to implement the model [51], [58]. The likelihood of the radio-tracking data is 0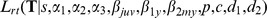.

### Likelihood for reproductive success data

We derived fecundity from the yearly counts of fledglings. The fecundity rate (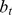) was defined as the number of offspring (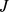) produced per mature female in year 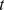. We assumed that 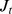 followed a Poisson distribution with parameter written as a product of the number of recorded reproducing females (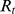) and fecundity rate (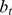), hence, 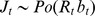. The likelihood of this sub-model is denoted as 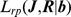.

### Likelihood for unmarked birds found dead

Within the state-space sub-model for population count data we included yearly counts of unmarked birds recovered dead by the local wildlife rescue centre. The estimated number of unmarked birds found dead by poisoning (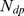) and by other causes (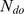) at time 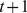 were modelled as drawn from a Multinomial distribution with sample size 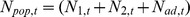 equal to the total number of individuals in the population (see [59] for the practical implementation of a Multinomial distribution with an unknown order 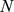), and a probability vector made up of the following probabilities
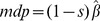
(6)

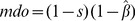
(7)

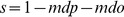
(8)where 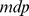 and 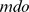 are the average probabilities (across age groups) of dying because of poisoning or other causes respectively, while the complementary probability with respect to one is the survival probability. 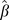 is the arithmetic mean of the age-specific proportion of deaths due to poisoning (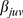, 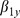, and 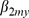).

We then assumed the total number of dead birds recovered in year 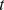 (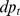 for poisoned and 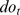 for other causes) to follow a Binomial distribution as
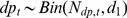
(9)

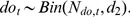
(10)The likelihood of unmarked birds found dead by poisoning was 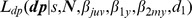, while the one for birds found dead by other causes was 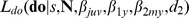.

### Likelihood of the integrated model

**Figure 6:**
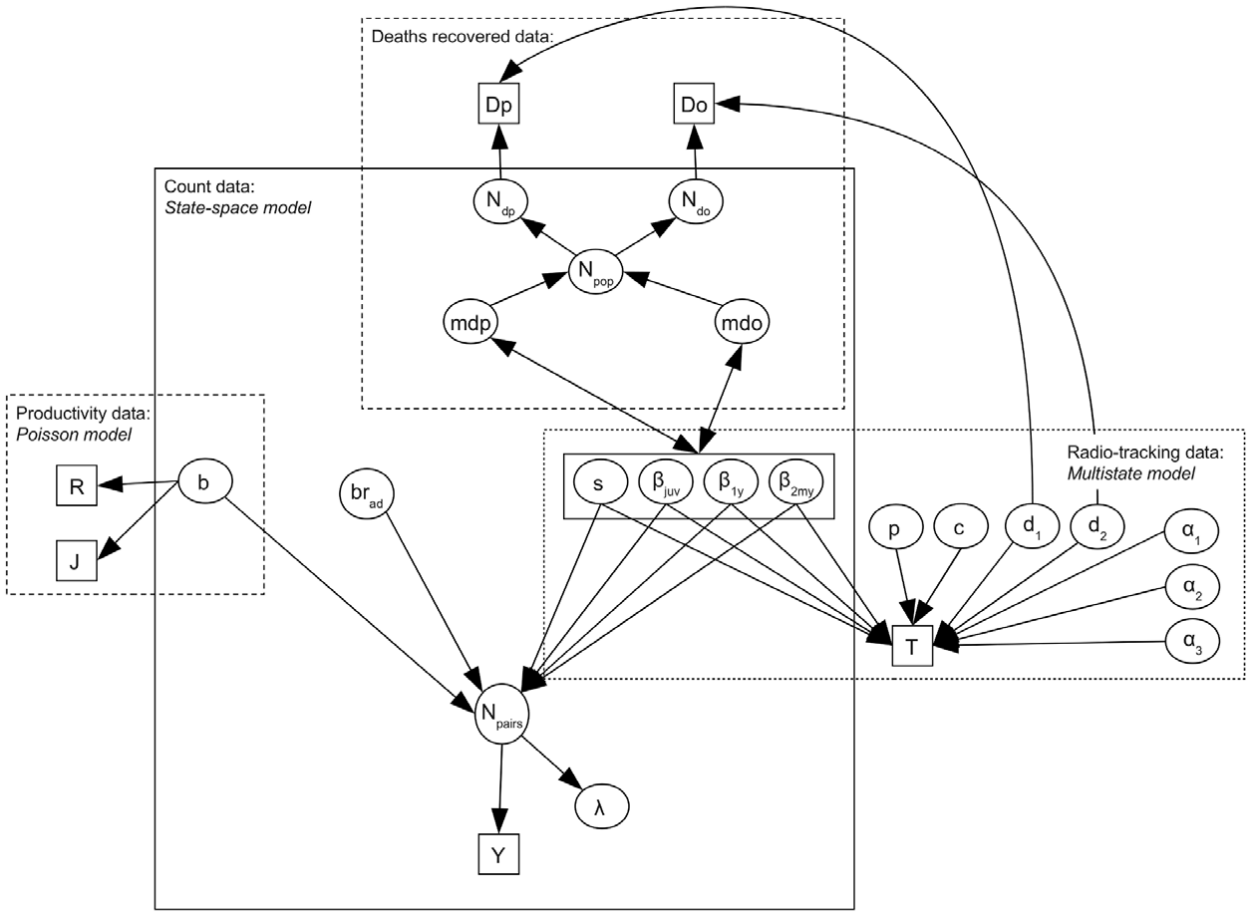
Graphical representation of the integrated population model. Data are symbolized by small rectangles, parameters by ellipses, the relationships between them by arrows and sub-models by open rectangles. Notation: 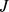: annual number of fledglings; 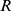: numbers of surveyed broods whose final fledging success was known; 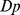: number of unmarked birds found dead by poisoning; 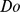: number of unmarked birds found dead by causes other than poisoning; 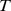: radio-tracking data; 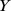: population count data; 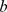: fecundity; 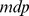: average probability, across age groups, of dying because of poisoning; 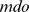: average probability, across age groups, of dying because of other causes; 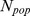: total number of individuals in the population; 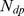: expected number of unmarked birds found dead by poisoning; 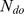: expected number of unmarked birds found dead by causes other than poisoning; 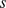: survival probability; 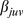: probability of death due to poisoning given that an animal died in its first year of life; 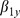: probability of death due to poisoning given that an animal died in its second year of life; 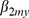: probability of death due to poisoning given that an animal died after its second year of life; 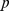: recapture probability of an animal with a functioning radio; 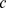: recapture probability of a radio-tagged animal which is alive but without an active radio signal; 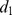: probability of encounter of a radio-tagged animal dead by poisoning but without an active radio signal; 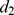: probability of encounter of a radio-tagged animal dead by other causes and without an active radio signal; 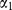, 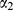, 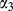: radio signal retention probability during the first three, the fourth and the fifth or more year of life, respectively; 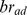: proportion of breeding females relative to the total number of females older than 2 years; 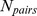: number of breeding females in the population; 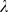: population growth rate. Priors are excluded from this graph to increase visibility.

The likelihoods of the four types of data have parameters in common, as displayed graphically in Fig. 6. By combining these data sources into a single analysis, and by using an integrated population model, more information can be used to estimate demographic parameters [55]. Assuming that the different data types are independent, the joint likelihood of the complete integrated model is the product of the different parts [16], [48], [60], thus
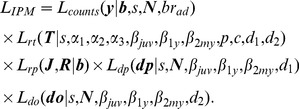
(11)Because the population was small, some individuals were likely to occur in different data sets, violating the assumption of independence between different likelihoods. However, a simulation study, which combined capture-recapture, population count and reproductive success data, showed that the violation of this assumption has only minimal impact on accuracy of parameter estimates [49]. Therefore, although the structure of our data slightly differ from such simulation study, we assumed a similarly minimal impact. The same assumption was employed in another recent study [52].

### Parameter estimation and model implementation

We used a hierarchical formulation of the integrated model to estimate temporal variability of fecundity (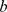), while keeping the other demographic rates constant over time. Thus, the annual estimates of fecundity were thought to originate from a random process with a common mean and a constant temporal variance. For the 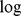 of this parameter we assumed
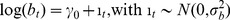
(12)where 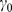 is the mean fecundity on the log scale and 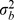 is the temporal variance of fecundity on the 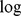 scale.

The joint likelihood of the model (eq. 11) is based on data of females only. However, we had also tracking data of males. These data were also included and modelled, but they contributed to the joint likelihood only improving the precision of parameter estimates that are all common in both sexes [55]. We used the Bayesian approach and Markov chain Monte Carlo (MCMC) simulation to estimate the parameters. We therefore based inference on the posterior distribution, which is proportional to the likelihood and the prior distribution. For the initial population sizes we used weakly informative priors [51], [52]. See Model Code S1 for the exact specification of the priors for all parameters. Some experimentation with different prior choices suggested they had low impact on the parameter estimates, indicating that the inferences were mainly determined by the information contained in the data. For the latent parameter 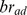 (proportion of breeding females older than 2 years), for which no explicit data were available, we specified three prior distributions to assess whether the integrated model provides an identifiable estimate of the parameter. For the latter, the posterior distribution was almost the same under the different sets of priors (a uniform distribution between 0.5 and 1 [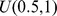], a normal distribution with mean 0.75 and variance 1000 truncated to the values between 0.5 and 1 [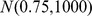
I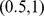], and a normal distribution with mean 0.5 and variance 0.25 truncated to the values between 0.5 and 1 [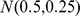
I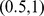]). Furthermore a trial integrated analysis with 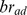 fixed to 0.8 [33] did not emphasize substantial changes in the other parameter estimates.

MCMC simulations were implemented in program WinBUGS [61], that we executed from R [62] with the package R2WinBUGS [63].

We ran three chains for 1,000,000 iterations of which we discarded the first 500,000 iterations as burn-in, and thinned the remaining every 20th sample for parameter estimation. We assessed the convergence of the MCMC simulations to the posterior distribution using the Brooks-Gelman-Rubin criterion, 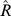, [64]. The 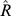 values were 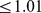 for all parameters by the end of the burn-in period. A 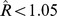 suggested that convergence may be assumed, and our burn-in period and run lengths were adequate [59]. Furthermore, the annual population growth rate (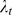) was estimated as a derived parameter, calculated as the ratio of the number of females in year 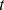 to the number of females in year 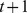. The growth rate averaged over the study period was calculated as the geometric mean of all year-specific values [53]. Then for each age class 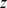, we derived the survival rate in the absence of illegal poisoning from the age-independent survival probability and the age-specific proportion of birds which died by poisoning (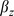) as
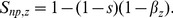
(13)One particularly useful feature of integrated models in the Bayesian framework is that predictions of the population sizes in the future can be made within the MCMC samples, thus fully accounting for all uncertainty in the parameter estimates [51]. We thus estimated population sizes for three further years (2011–2013) after the last one for which real data were available. A 3-year time interval reflects the typical duration of the decision-making processes related to management actions, and allows estimation of future population sizes while avoiding excessive increases of uncertainty.

We assessed the magnitude of the improvements in the estimates of demographic parameters by comparing the precision (standard error and 95% credible interval) of these estimates obtained from (i) a stand-alone multi-state model (MS) including only radio-tracking data, (ii) an Integrated Population Model including all data sets but those referred to unmarked birds found dead on the local rehabilitation centre (IPM1), and (iii) a full model based on all data sets available (IPM2).

### Modelling the effect of poisoning on population growth rate

To assess the demographic consequences of poison related mortality we used a deterministic Leslie matrix population model (as defined in eq. 1) with parameter estimates obtained from the full integrated model (IPM2, Table 1), to simulate the demography for different levels of poisoning, all other things being equal. We thus used perturbation analysis to compute the sensitivity and elasticity of the population growth rate to different demographic parameters and the proportional decrease in survival due to illegal poisoning. The latter was calculated, for the 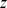-th age class, as 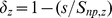. In its standard form, the sensitivity measures the impact of changes in matrix elements (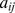) on population growth rate (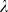):
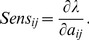
(14)Sensitivities can also be applied to the vital rates (low-level parameters) [4]. This is done by tracking the changes in 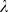 resulting from changes in the vital rates implicit in the matrix elements 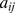.

Similarly, elasticity values can also be calculated for vital rates. Standard elasticity considers the proportional change in 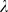 due to a proportional change in a parameter:
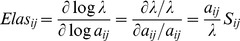
(15)where 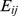 is the elasticity of the matrix element 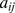, and 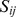 is its sensitivity. In analogy, elasticity values of vital rates can be obtained by multiplying vital rate sensitivity by 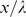, where 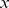 is the value of the vital rate under consideration [4]. Unlike the values for matrix elements, vital rates sensitivity and elasticity may be negative, but as it is the magnitude of the change that is of interest, rather than its sign, absolute values are quoted throughout the paper. Note that the elasticities of matrix cells sum to 1, but those for all matrix elements do not [4].

## Supporting Information

Figure S1
**Transition matrix for the multi-state sub-model.** From the state at 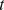 (rows) to state at 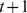 (columns) different transition probabilities could encompass the following probabilities: annual survival (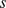), radio signal retention during the first three, the fourth and the fifth or more year of life (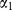, 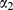, and 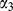, respectively), and mortality due to poisoning given that an animal has died during its first, second or more than second year of life (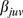, 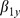, and 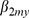 respectively). State abbreviations are a combination of a prefix referred either to the six age-classes (from ‘juv’, for juveniles, to ‘5my’ for 5 or more year olds) or to the cause of death (‘DP’ for dead by poison, ‘DO’ for dead by other reasons), and a suffix that specifies the presence of a functioning radio (‘.t’ and ‘.nt’ for with and without radio signal, respectively).(TIF)

Figure S2
**Observation matrix for the multi-state sub-model.** The matrix specifies the probability of each event (in column, coded with numbers from 1 to 16) conditional on each state (rows). Codes from ‘1’ to ‘6’ refer to encounters of individuals alive with a functioning radio and belonging to one of the six age-classes, from juvenile to 5 or more years old birds. Codes from ‘9’ to ‘13’ refer to birds alive and without a functioning radio. Codes ‘7’ and ‘14’ refer to individuals found poisoned with and without a functioning radio respectively, while ‘8’ and ‘15’ code for birds found dead for causes other than poisoning, with and without a functioning radio respectively. Code ‘16’ refers to cases when the radio signal cannot be heard and the animal cannot be seen. 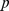 is the probability of encounter of an animal with a functioning radio, 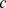 is the probability of encounter of an animal alive without an active radio signal, 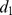 is the probability of encounter of an animal dead by poisoning and without an active radio signal, 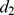 is the probability of encounter of an animal dead by other causes and without an active radio signal. For state abbreviations see transition matrix in Fig. S1.(TIF)

Model Code S1 **R and WinBUGS codes including the data to fit the integrated population model.** The directories in the code need to be customized.
(R) Click here for additional data file.
