# Supplementary figures and images for: Demographic Consequences of Poison-Related Mortality in a Threatened Bird of Prey
Source: PLoS One. 2012 Nov 14;7(11):e49187. doi: 10.1371/journal.pone.0049187 (PMC3498344; doi:10.1371/journal.pone.0049187)

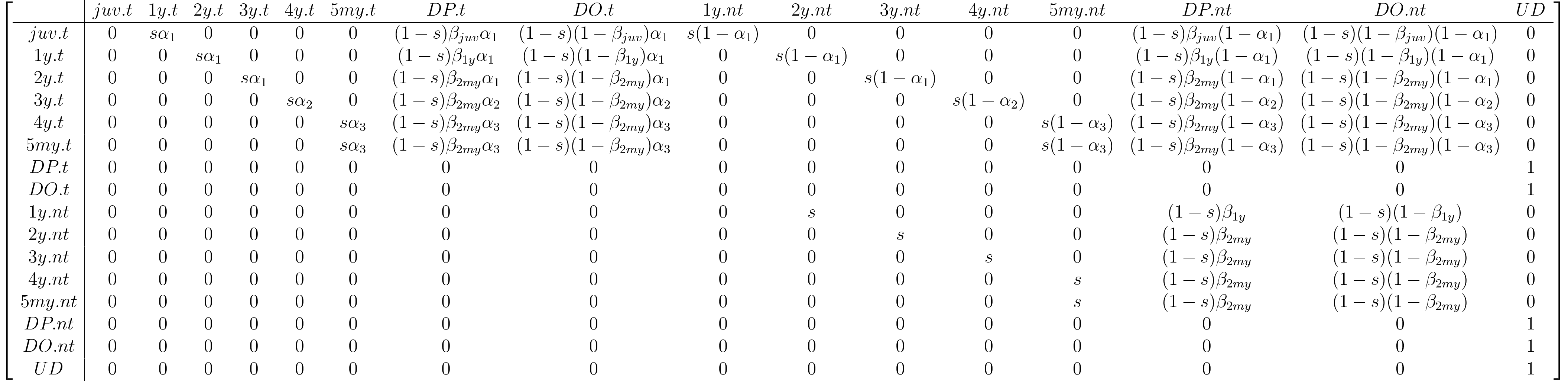

Supplement: Figure S1 — Transition matrix for the multi-state sub-model. From the state at (rows) to state at (columns) different transition probabilities could encompass the following probabilities: annual survival (), radio signal retention during the first three, the fourth and the fifth or more year of life (, , and , respectively), and mortality due to poisoning given that an animal has died during its first, second or more than second year of life (, , and respectively). State abbreviations are a combination of a prefix referred either to the six age-classes (from ‘juv’, for juveniles, to ‘5my’ for 5 or more year olds) or to the cause of death (‘DP’ for dead by poison, ‘DO’ for dead by other reasons), and a suffix that specifies the presence of a functioning radio (‘.t’ and ‘.nt’ for with and without radio signal, respectively). (TIF) [file pone.0049187.s001.tif]

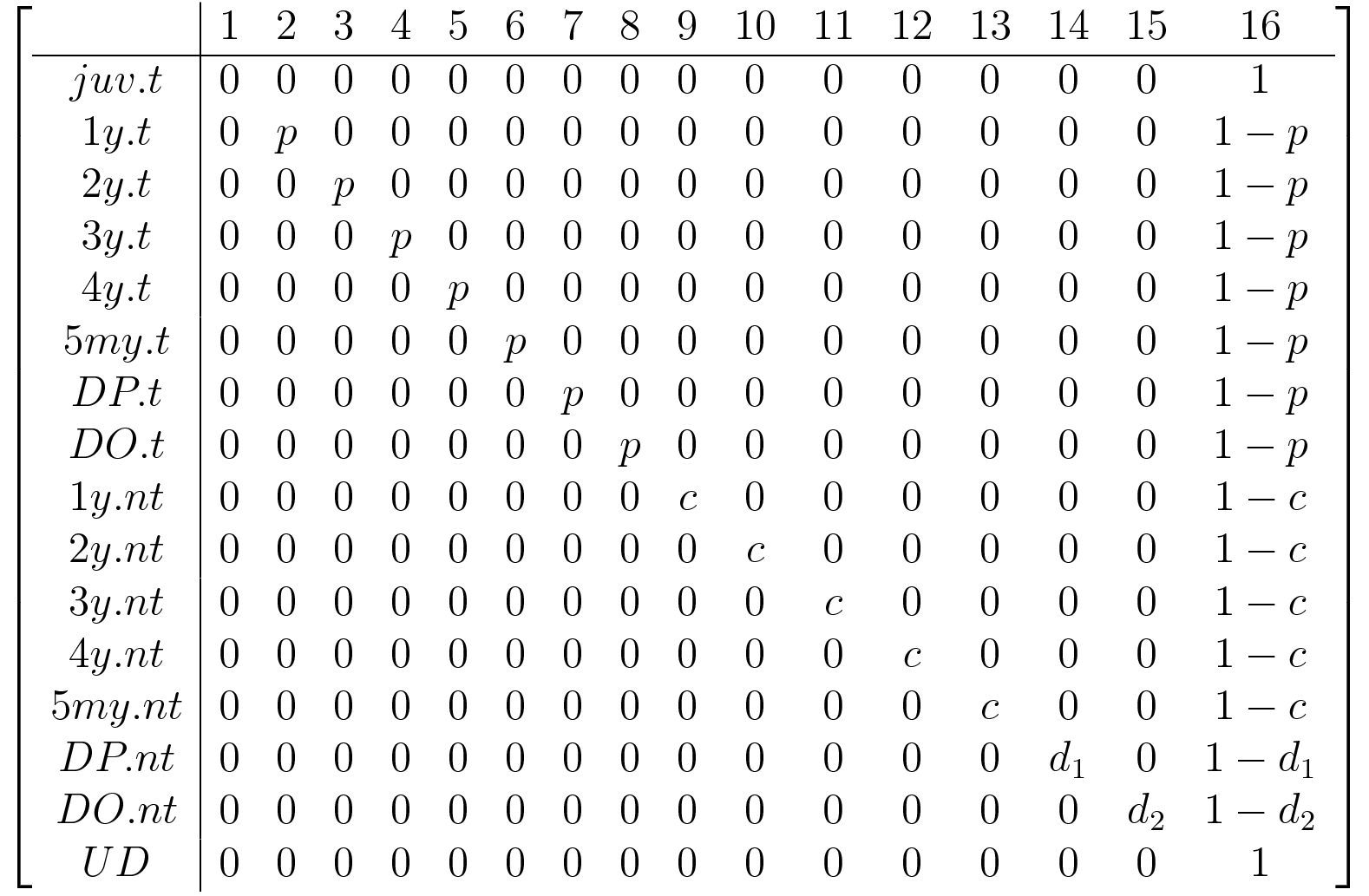

Supplement: Figure S2 — Observation matrix for the multi-state sub-model. The matrix specifies the probability of each event (in column, coded with numbers from 1 to 16) conditional on each state (rows). Codes from ‘1’ to ‘6’ refer to encounters of individuals alive with a functioning radio and belonging to one of the six age-classes, from juvenile to 5 or more years old birds. Codes from ‘9’ to ‘13’ refer to birds alive and without a functioning radio. Codes ‘7’ and ‘14’ refer to individuals found poisoned with and without a functioning radio respectively, while ‘8’ and ‘15’ code for birds found dead for causes other than poisoning, with and without a functioning radio respectively. Code ‘16’ refers to cases when the radio signal cannot be heard and the animal cannot be seen. is the probability of encounter of an animal with a functioning radio, is the probability of encounter of an animal alive without an active radio signal, is the probability of encounter of an animal dead by poisoning and without an active radio signal, is the probability of encounter of an animal dead by other causes and without an active radio signal. For state abbreviations see transition matrix in Fig. S1. (TIF) [file pone.0049187.s002.tif]
